# Development and validation of an interpretable machine learning for mortality prediction in patients with sepsis

**DOI:** 10.3389/frai.2024.1348907

**Published:** 2024-07-08

**Authors:** Bihua He, Zheng Qiu

**Affiliations:** ^1^Department of Neurology, Third People's Hospital of Hubei Province, Wuhan, China; ^2^Department of Neurology, Hubei NO. 3 People’s Hospital of Jianghan University, Wuhan, China

**Keywords:** Sepsis, machine learning, mortality, prediction, database

## Abstract

**Introduction:**

Sepsis is a leading cause of death. However, there is a lack of useful model to predict outcome in sepsis. Herein, the aim of this study was to develop an explainable machine learning (ML) model for predicting 28-day mortality in patients with sepsis based on Sepsis 3.0 criteria.

**Methods:**

We obtained the data from the Medical Information Mart for Intensive Care (MIMIC)-III database (version 1.4). The overall data was randomly assigned to the training and testing sets at a ratio of 3:1. Following the application of LASSO regression analysis to identify the modeling variables, we proceeded to develop models using Extreme Gradient Boost (XGBoost), Logistic Regression (LR), Support Vector Machine (SVM), and Random Forest (RF) techniques with 5-fold cross-validation. The optimal model was selected based on its area under the curve (AUC). Finally, the Shapley additive explanations (SHAP) method was used to interpret the optimal model.

**Results:**

A total of 5,834 septic adults were enrolled, the median age was 66 years (IQR, 54–78 years) and 2,342 (40.1%) were women. After feature selection, 14 variables were included for developing model in the training set. The XGBoost model (AUC: 0.806) showed superior performance with AUC, compared with RF (AUC: 0.794), LR (AUC: 0.782) and SVM model (AUC: 0.687). SHAP summary analysis for XGBoost model showed that urine output on day 1, age, blood urea nitrogen and body mass index were the top four contributors. SHAP dependence analysis demonstrated insightful nonlinear interactive associations between factors and outcome. SHAP force analysis provided three samples for model prediction.

**Conclusion:**

In conclusion, our study successfully demonstrated the efficacy of ML models in predicting 28-day mortality in sepsis patients, while highlighting the potential of the SHAP method to enhance model transparency and aid in clinical decision-making.

## Introduction

Sepsis is a common life-threatening condition associated with high morbidity and mortality (Singer et al., 2016). Data from the Global Burden of Diseases (GBD) project show that in 2017, there were an estimated 48.9 million sepsis cases occurred worldwide, with approximately 11.0 million sepsis-related deaths (Rudd et al., 2020). Despite the advancement of various treatments of sepsis, the mortality remains unacceptably high (Evans et al., 2021).

Monitoring and providing early warning are essential steps in the sepsis process (Shamout et al., 2020). Evidence from a multicenter randomized controlled trial showed that using of an automated predictive model to identify hospitalized patients at high risk for clinical deterioration was associated with decreased mortality (Escobar et al., 2020). Machine learning (ML) is a subfield of artificial intelligence, which enables complex decision-making in clinical practice. Previous studies have demonstrated that ML is superior to traditional predictive models, such as Cox regressions and Logistic regression (Hu et al., 2022a,b,c).

In the context of sepsis outcome prediction, previous studies have employed fuzzy methods to enhance interpretability. For instance, fuzzy decision-making approaches have been utilized to derive transparent decision rules based on expert knowledge and patient data (Yang et al., 2023). Probabilistic fuzzy systems have been used to model uncertainties and capture the relationships between input variables and sepsis mortality outcomes. Multi-stage modeling using fuzzy multi-criteria feature selection has been employed to identify relevant features and interpret their contributions to the prediction model. Fuzzy modeling techniques have been utilized to develop interpretable and transparent models for sepsis prognosis. These fuzzy-based approaches emphasize the interpretability of the models by providing transparent rules, linguistic variables, and membership functions that can be easily understood and validated by domain experts.

In order to conquer the black box problem, Lundberg and colleagues recently proposed a Shapley additive explanations (SHAP) method (Lundberg et al., 2020). This explainable method has been successfully applied to interpret the ML models in predicting mortality among patients with acute kidney injury (Hu et al., 2022c) and identifying patients at risk of reattendance at discharge from emergency departments (Chmiel et al., 2021). However, the study to predict outcome using explainable ML approach in patients with sepsis remains scarce.

Therefore, combinatory uses of ML methods and SHAP method for prognosis prediction in the context of sepsis are worth exploring. In this investigation, we aimed to develop an explainable machine learning (ML) model to predict 28-day mortality in patients admitted to the intensive care unit (ICU) with a diagnosis of sepsis.

## Methods

### Data source and ethical approval

The data for this study were obtained from the Medical Information Mart for Intensive Care (MIMIC)-III database (version 1.4). The MIMIC-III is a large, freely available database, which contains comprehensive information on more than 60,000 intensive care unit (ICU) admissions at the Beth Israel Deaconess Medical Center (Boston, Mass.), between 2001 and 2012 (Johnson et al., 2016). This database contains highly granular data, including demographic characteristics, vital signs, laboratory results, treatments and clinical outcomes.

The MIMIC-III project was approved by the ethics committees of the Massachusetts Institute of Technology (United States) and Beth Israel Deaconess Medical Center (United States), and one author in this study has been approved to get access to the MIMIC-III database after completing the Protecting Human Research Participants examination. This database is a public de-identified database thus informed consent and approval of the Institutional Review Board was waived. Written informed consent was obtained from individual at ICU admission. All procedures performed in this study involving human participants were in accordance with the ethical standards of the institutional and national research committee and with the 2013 Helsinki declaration (World Medical Association, 2013).

### Study population and outcome

Inclusion criteria was adult patients (> 18 years) with a diagnosis of sepsis in accordance with the Third International Consensus Definitions for Sepsis (Sepsis-3) (Singer et al., 2016), which involved the acquisition of a blood culture (test for infection) contemporaneous to administration of antibiotics and combined with a change in Sequential Organ Failure Assessment (SOFA) score ≥ 2 on day 1 after ICU admission (Supplementary Figure S1). The SOFA score is a widely used tool in critical care medicine to assess the severity of organ dysfunction in critically ill patients, including those with sepsis. It provides a quantifiable measure of the extent of organ dysfunction based on several physiological parameters. The SOFA score evaluates six different organ systems: respiratory, cardiovascular, hepatic, coagulation, renal, and neurological. Each organ system is assigned a score based on the patient’s clinical measurements and laboratory values. The scores from each organ system are then summed to provide an overall SOFA score. Patients who met the following criteria were excluded: (1) multiple ICU admission (only the first admission per patient was analyzed); (2) ICU length of stay <24 h. The primary outcome was death from any cause at 28 days after ICU admission, which included both in-hospital and post-hospital mortality up to 28 days. In MIMIC-III database, out of hospital mortality is obtained using the Social Security Administration Death Master database and in-hospital mortality is sourced from the hospital database. The 28-day period is considered significant in sepsis because it reflects the acute phase of the illness and is a critical window for patient outcomes. It assists healthcare providers in determining the need for intensive care resources, including bed availability, staffing, and other supportive measures during this crucial timeframe (Peng et al., 2023).

### Data extraction and preprocessing

We extracted demographic characteristics, comorbidities, vital signs, laboratory results and severity scores from the MIMIC-III database using Structured Query Language (SQL) and PostgreSQL software (version 9.6.22). In brief, the demographic characteristics included age, gender and body mass index (BMI). The comorbidities included hypertension, diabetes, congestive heart failure, renal failure, liver disease, tumor and rheumatoid arthritis. The vital signs included heart rate, respiratory rate, systolic blood pressure, diastolic blood pressure, mean artery pressure, body temperature, SpO_2_. We extracted the mean values for the vital signs because they are measured repeatedly and could be greatly affected by the external environment. For laboratory results, we selected the maximum value for the following variables: glucose, lactate, white blood cell count, aspartate aminotransferase, alanine aminotransferase, creatine kinase MB, lactate dehydrogenase, total bilirubin, prothrombin time, partial thromboplastin time, international normalized ratio, creatinine, blood urea nitrogen (BUN), sodium, chloride, potassium, bicarbonate, and anion gap. Furthermore, we selected the minimum value for the following variables: platelet, hematocrit, hemoglobin, and albumin. The Logistic Organ Dysfunction System (LODS) (Le Gall et al., 1996) and Sequential Organ Failure Assessment (SOFA) (Raith et al., 2017) score were recalculated with the maximum value according to their components on day 1. Moreover, we included total urine output on the first day of the ICU. All the data were collected on day 1 after ICU admission. To avoid overfitting, we excluded LODS score and SOFA score for model development (Supplementary Table S1). The code and data extraction details can be found in the repository available at GitHub MIT-LCP/mimic-code. The components of the SOFA score include: (1) respiratory: PaO2/FiO2 ratio (partial pressure of arterial oxygen/fraction of inspired oxygen); (2) coagulation: platelet count or international normalized ratio (INR); (3) liver: bilirubin level; (4) cardiovascular: mean arterial pressure or use of vasopressors; (5) central nervous system: Glasgow Coma Scale score; (6) renal: Serum creatinine or urine output. Each component is assigned a score from 0 to 4, with higher scores indicating more severe dysfunction. The total SOFA score is the sum of the scores from all the components (Supplementary file). The components of the LODS score include: (1) cardiovascular: mean arterial pressure, use of vasopressors, or the need for cardiac massage; (2) respiratory: oxygenation index and positive end-expiratory pressure; (3) renal: serum creatinine level and urine output; (4) hematologic: platelet count and white blood cell count; (5) hepatic: bilirubin level; (6) neurologic: Glasgow Coma Scale score. Similar to the SOFA score, each component is assigned a score, and the total LODS score is calculated by summing these scores. The LODS is typically used for the assessment of organ dysfunction in critically ill patients, including those with sepsis (Supplementary file).

In this study, the selection of maximum or minimum values for specific variables was driven by the clinical context and their relevance to the research question under investigation. Our primary objective was to capture the extreme values of these variables, as they hold utmost importance in clinical decision-making and bear significant implications for patient outcomes. Variables such as glucose, lactate, white blood cell count, aspartate aminotransferase, and alanine aminotransferase serve as common markers for metabolic dysfunction, inflammation, and organ damage. Higher values of these variables often suggest the presence of pathology or indicate the severity of a disease, thereby signifying critical conditions or disease progression. On the other hand, variables like platelet count, hematocrit, hemoglobin, and albumin have specific optimal or desirable ranges. Values falling below the normal range for these variables may indicate conditions such as anemia or nutritional deficiencies, which can impact overall health.

**Figure 1:**
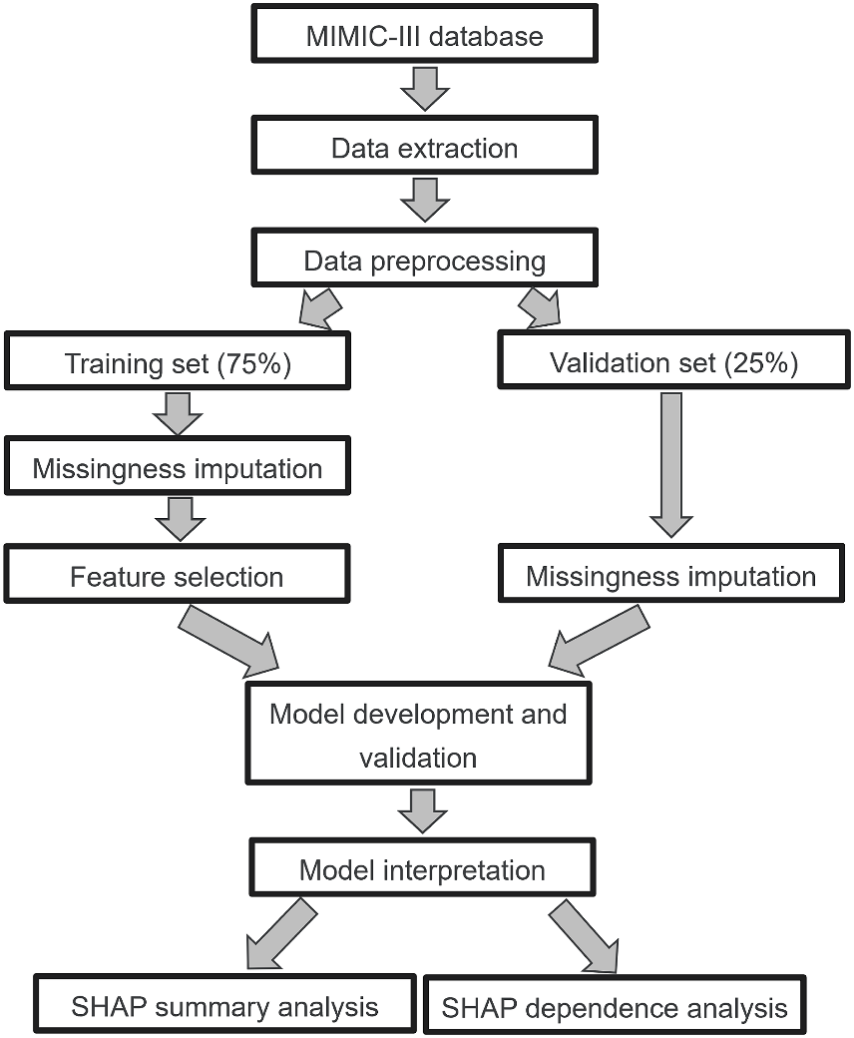
Study design. MIMIC-III, Medical Information Mart for Intensive Care III; LASSO, least absolute shrinkage and selection operator; SHAP, Shapley additive explanation.

We performed the analyses in accordance with the published studies (Tseng et al., 2020; Hu et al., 2022b). First, we defined outlier as a data point that falls below [first quartile (Q1) - 1.5* interquartile range (IQR)] or above [third quartile (Q3) + 1.5*IQR]. It defines a range beyond which data points are considered potential outliers. Data points falling below (Q1–1.5*IQR) or above (Q3 + 1.5*IQR) are typically flagged as outliers. Then we dropped these outliers (Korhonen et al., 2024). Second, we removed those features with greater than 50% missing values. The fraction of missing values for all features are presented in Supplementary Figure S4. Third, all septic individuals were split into a training (*N* = 4,375) set and a validation (*N* = 1,459) set using computer-generated random numbers at a ratio of 3:1. After data splitting, Multiple Imputation by Chained Equations (MICE) was carried out independently for both the training set and the testing set (Zhang, 2016). This approach entails treating the training and testing sets as separate entities during the data imputation process, ensuring no information exchange between them. Furthermore, we used a least absolute shrinkage and selection operator (LASSO) regression with 5-fold cross-validation to select the features in the training set (Supplementary Figure S5). The LASSO is a well-known method that could mitigate overfitting during feature selection (Bloniarz et al., 2016). The full details for data preprocessing are presented in Figure 1, which including data extraction, data preprocessing, model development and validation, model interpretable.

### Model development and validation

First, we evaluated multiple candidate prediction models, including extreme gradient boost (XGBoost), logistic regression (LR), support vector machines (SVM), and Random Forest (RF), to predict 28-day mortality among patients with sepsis in the training set. XGBoost, known for its optimized implementation of gradient boosting, demonstrated high bias and low variance. LR was chosen as the baseline model due to its linear nature. SVM utilized the boundary hyperplane between positive and negative samples for prediction, while RF employed an ensemble of decision trees to reduce prediction errors caused by individual trees.

Next, we incorporated feature selection, model selection, and hyperparameter tuning into the process. For feature selection, LASSO regression was employed to identify relevant features. Model selection involved comparing the performance of the candidate models (XGBoost, LR, SVM, RF) using appropriate evaluation metrics. Hyperparameter tuning was performed to optimize the parameters of each model. Specifically, the hyperparameters of the XGBoost model were fine-tuned using Scikit-learn’s GridSearchCV in combination with 5-fold cross-validation (Khan et al., 2020). The selected hyperparameters for optimization included learning_rate, gamma, max_depth, subsample, min_child_weight, and n_estimators. Similarly, the GridSearchCV method from scikit-learn was utilized to optimize the hyperparameters of other models (SVM, RF, LR), while also employing 5-fold cross-validation.

Lastly, the final selected model is assessed by evaluating its generalization performance on the test set. AUC is calculated using the selected features on the test set, thereby validating the model’s performance.

### Model explainability

Since SHAP utilizes game theory to transform the model into a sum effect of all feature attributes, enabling the calculation of the impact of each feature on the final prediction, we proposed three analytical approaches: SHAP summary analysis, SHAP dependence analysis, and SHAP force analysis. These approaches aimed to provide a comprehensive understanding of the optimal model at both the feature level and individual level.

### Statistical analysis

For continuous variables, the mean [standard deviation (SD)] and median [interquartile range (IQR)] were used for normally and abnormally distributed data, respectively. Categorical variables were expressed as absolute values along with percentages. For the comparison of participants with survivors and non-survivors, *χ*^2^ test, Fisher’s exact test, Student’s *t* test or Mann–Whitney U test were used when appropriate. Spearman’s rank correlation coefficient was used to assess association between the candidate features.

In this study, an outlier is defined as a data point that falls below (Q1–1.5*IQR) or above (Q3 + 1.5*IQR), and then we dropped these outliers using R statistical software. Additionally, we provided the related hyperparameters (values that control the machine learning process) for each machine learning model (Supplementary Table S4).

To evaluate the predictive performance of the four models, we calculated eight representative performance evaluation measures: area under the receiver operator characteristic curve (AUC), cut-off, accuracy, sensitivity, specificity, positive predictive value (PPV), negative predictive value (NPV) and F1 score. In our study, we utilized the Youden index to select the optimal cut-off point for determining the predictive threshold of models. The Youden index is a commonly used metric that maximizes the sum of sensitivity and specificity, providing an optimal balance between the two (Chen et al., 2015). Additionally, the cut-off values represent the threshold values used to classify individuals into the two categories: survivors and non-survivors. We evaluated the calibration of the prediction model by conducting the Hosmer-Lemeshow goodness-of-fit test, calculating the Brier score and plotting the calibration curve. The SHAP summary analysis, SHAP dependence analysis and SHAP force analysis were applied to describe the impacts of the feature values on the optimal model, respectively.

We performed statistical analyses using the sklearn machine learning package (version 0.24.2), xgboost package (version 1.5.0), and shap package (version 0.40.0) in Python (version 3.6.6, Python Software Foundation, Wilmington, DE, United States) and R (version 3.6.1, Project for Statistical Computing, Vienna, Austria) software. A two-sided *p* < 0.05 was considered statistically significant.

## Results

### Characteristics of study participants

Of 61,532 admissions from the MIMIC-III database, 7,924 were diagnosed with sepsis in the first day after ICU admission. 2090 patients were excluded in our study because of multiple ICU admission (*N* = 1,610) and ICU length of stay <24 h (*N* = 480). A total of 5,834 patients with sepsis were then enrolled. The screening flowchart is presented in Supplementary Figure S2. Among 5,834 septic patients, the median age was 66 years (IQR, 54–78 years), 2,342 (40.1%) were women, and the 28-day all-cause mortality was 15.5% (903/5834). The top three comorbidities were diabetes (1,557/5834, 26.7%), congestive heart failure (878/5834, 15.0%) and renal failure (802/5834, 13.7%). Additional participant characteristics are presented in Table 1.

**Table 1:** Baseline characteristics between survivors and non-survivors.

Characteristics	Total (*N* = 5,834)	Survivors (*N* = 4,931)	Non-survivors (*N* = 903)	*P* value
**Demographics**				
Age, year	66 (54–78)	65 (53–77)	73 (60–82)	<0.001
Gender				0.016
Male, *n* (%)	3,492 (59.9)	2,984 (60.5)	508 (56.3)	
Female, *n* (%)	2,342 (40.1)	1947 (39.5)	385 (43.7)	
Body mass index, kg/m^2^	28 (24–32)	28 (24–32)	26 (23–30)	<0.001
**Comorbidities**				
Diabetes, *n* (%)	1,557 (26.7)	1,324 (26.9)	233 (25.8)	0.513
Congestive heart failure, *n* (%)	878(15.0)	662 (13.4)	216 (23.9)	<0.001
Renal failure, *n* (%)	802 (13.7)	643 (13.0)	159 (17.6)	<0.001
Hypertension, *n* (%)	697 (11.9)	571 (11.6)	126 (14.0)	0.043
Liver disease, *n* (%)	377 (6.5)	287 (5.8)	90 (10.0)	<0.001
Tumor, *n* (%)	369 (6.3)	264 (5.4)	105 (11.6)	<0.001
Rheumatoid arthritis, *n* (%)	153 (2.6)	122 (2.5)	31 (3.4)	0.097
**Vital signs on day 1**				
Heart rate, bpm	104 (91–118)	103 (90–117)	109 (94–126)	<0.001
Systolic blood pressure, mmHg	89 (79–99)	89 (81–99)	84 (73–95)	<0.001
Diastolic blood pressure, mmHg	43 (37–50)	44 (38–50)	40 (33–48)	<0.001
Mean arterial pressure, mmHg	57 (51–64)	58 (52–64)	55 (47–62)	<0.001
Respiratory rate	27 (23–31)	26 (23–30)	29 (24–33)	<0.001
Body temperature, °C	37.6 (37.1–38.2)	37.6 (37.1–38.1)	37.6 (36.9–38.2)	0.011
SpO_2_, %	93 (90–95)	93 (91–95)	92 (88–95)	<0.001
**Laboratory findings on day 1**				
Blood glucose, mg/dL	170 (139–212)	168 (139–207)	179 (143–243)	<0.001
Lactate, mmol/L	2.3 (1.5–3.5)	2.3 (1.5–3.3)	2.6 (1.6–4.6)	<0.001
White blood cell count, ×10^3^/uL	13.6 (10.1–18.2)	13.3 (10.1–17.8)	15.1 (10.3–20.4)	<0.001
Platelets, ×10^3^/uL	170 (119–232)	169 (121–229)	175 (109–248)	0.996
Hematocrit, %	29 (25–33)	29 (25–33)	30 (26–34)	<0.001
Hemoglobin, g/dL	9.8 (8.4–11.3)	9.7 (8.4–11.2)	10.0 (8.7–11.4)	<0.001
Total bilirubin, mg/dL	0.7 (0.4–1.5)	0.7 (0.4–1.4)	0.8 (0.5–2.1)	<0.001
Prothrombin time, s	15 (13–17)	15 (13–17)	16 (14–20)	<0.001
Partial thromboplastin time, s	33 (28–45)	33 (28–43)	35 (28–57)	<0.001
International normalized ratio	1.3 (1.2–1.6)	1.3 (1.2–1.5)	1.4 (1.2–2.0)	<0.001
Creatinine, mg/dL	1.1 (0.8–1.6)	1.0 (0.8–1.5)	1.4 (0.9–2.5)	<0.001
Blood urea nitrogen, mg/dL	21 (15–35)	20 (14–31)	32 (21–51)	<0.001
Sodium, mmol/L	141 (138–143)	141 (138–143)	141 (137–144)	0.509
Chloride, mmol/L	109 (105–112)	109 (105–112)	107 (102–112)	<0.001
Potassium, mmol/L	4.6 (4.2–5.3)	4.6 (4.2–5.3)	4.6 (4.1–5.2)	0.112
Bicarbonate, mmol/L	25 (22–27)	25 (23–27)	24 (21–27)	<0.001
Anion gap	15 (12–18)	14 (12–17)	17 (14–21)	<0.001
Urine output on day 1, mL	1723 (1090–2,540)	1818 (1205–2,630)	1,097 (606–1820)	<0.001
**Severity of illness scores**				
GCS	15 (14–15)	15 (14–15)	15 (12–15)	0.561
SOFA	5 (3–7)	5 (3–6)	6 (4–9)	<0.001
LODS	4 (3–7)	4 (3–6)	7 (4–9)	<0.001

Data were reported as no. (%) or median (IQR).

GCS, glasgow coma scale; SOFA, sequential organ failure assessment; LODS, logistic organ dysfunction system.

Univariate comparison of two groups revealed that non-survivors were older [73 (60–82) vs. 65 (53–77) years, *p* < 0.001], and had lower body mass index [26 (23–30) vs. 28 (24–32) kg/m^2^, *p* < 0.001], compared with survivors. Non-survivors were more likely to have underlying comorbidities, including congestive heart failure (23.9% vs. 13.4%, *p* < 0.001), renal failure (17.6% vs. 13.0%, *p* < 0.001), liver disease (10.0% vs. 5.8%, *p* < 0.001) and tumor (11.6% vs. 5.4%, *p* < 0.001). Additionally, survivor patients vs. non-survivors had lower severity of illness scores, included SOFA score [6 (4–9) vs. 5 (3–6), *p* < 0.001] and LODS score [7 (4–9) vs. 4 (3–6), *p* < 0.001]. The distribution plot of mortality days is shown in Supplementary Figure S3, we observe a high mortality rate within the first 7 days after ICU admission, this early peak in mortality aligns with the well-established understanding of sepsis as a rapidly progressing and life-threatening condition.

### Feature selection

The overall study design is displayed in Figure 1. We extracted 41 clinical variables from the MIMIC-III database, including demographic characteristics, comorbidities, vital signs, laboratory results and severity scores (Supplementary Table S1). Five variables were removed because of missingness >50%. The fraction of missing values for all variables is presented in Supplementary Figure S4. After randomly splitting, there were 4,375 patients (75%) in the training set and 1,459 patients (25%) in the testing set. Then, we applied a classical LASSO regression involved using 5-fold cross-validation with the minimum criteria in the training set (Supplementary Figure S5). A total of 14 variables were finally included for our analysis (Supplementary Table S2). Moreover, we tested the association of these variables by calculating Spearman’s rank correlation coefficient (Supplementary Figure S6). The highest correlations were observed between anion gap and lactate (r = 0.5) and anion gap and blood urea nitrogen (r = 0.5).

### Model development and validation

**Figure 2:**
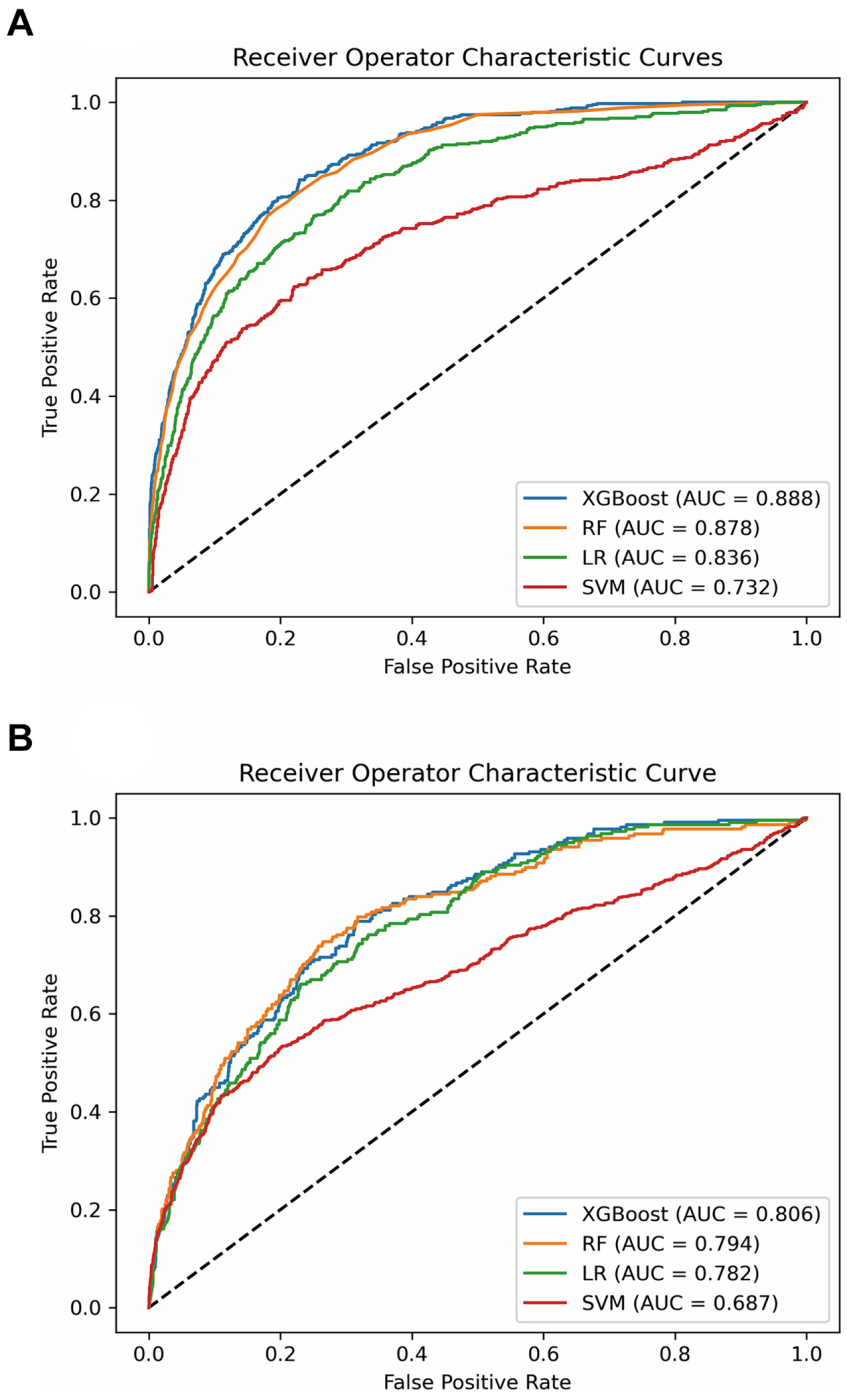
ROC analysis for the four models. **(A)** Comparison of AUC values between the four models in the training set. **(B)** Comparison of AUC values between the four models in the testing set. ROC, receiver operating characteristic curve; AUC, area under the curve; XGBoost, extreme gradient boost; RF, random forest; LR, logistic regression; SVM, support vector machine.

We found that all of the variables between the training set (*N* = 4,375) and the verification set (*N* = 1,459) did not exhibit statistically significant differences (all *p* > 0.05) (Supplementary Table S3). We developed XGBoost, RF, LR and SVM models with 5-fold cross-validation technique. The results are presented in Table 2. We found that the XGBoost model (AUC: 0.806) had superior performance with AUC, compared with RF (AUC: 0.794), LR (AUC: 0.782) and SVM model (AUC: 0.687) (Figure 2 and Table 2). To evaluate the predictive performance of the XGBoost model, we also added LODS score and SOFA score as the reference standard for prediction of the outcome in sepsis. The AUCs for LODS and SOFA were 0.728 and 0.685, respectively (Supplementary Figure S7). Additionally, we used the Hosmer-Lemeshow test and Brier score to assess the calibration for the XGBoost model, the results showed that the Chi-square value was 8.216 (*p* = 0.354) and the Brier score was 0.05, respectively (Supplementary Figure S8). Meanwhile, the calibration curve also demonstrated a good agreement between the predicted and observed values for the XGBoost model (Supplementary Figure S8). Therefore, the XGBoost model was identified as the optimal model in this study.

**Table 2:** Comparisons of performance between the four models in the testing set.

Models	AUC	Cut-off	Accuracy	Sensitivity	Specificity	PPV	NPV	F1
XGBoost	0.806	0.186	0.855	0.761	0.701	0.577	0.864	0.373
RF	0.794	0.121	0.846	0.763	0.690	0.188	0.873	0.291
LR	0.782	0.231	0.843	0.732	0.695	0.442	0.881	0.429
SVM	0.687	0.168	0.832	0.621	0.616	0.216	0.913	0.271

AUC, area under the curve; ML, machine learning; XGBoost, extreme gradient boost; LR, logistic regression; SVM, support vector machines; RF, random forest; LODS, logistic organ dysfunction system; SOFA, sequential organ failure assessment; PPV, positive predictive value; NPV, negative predictive value.

### Model interpretability at the feature level

**Figure 3:**
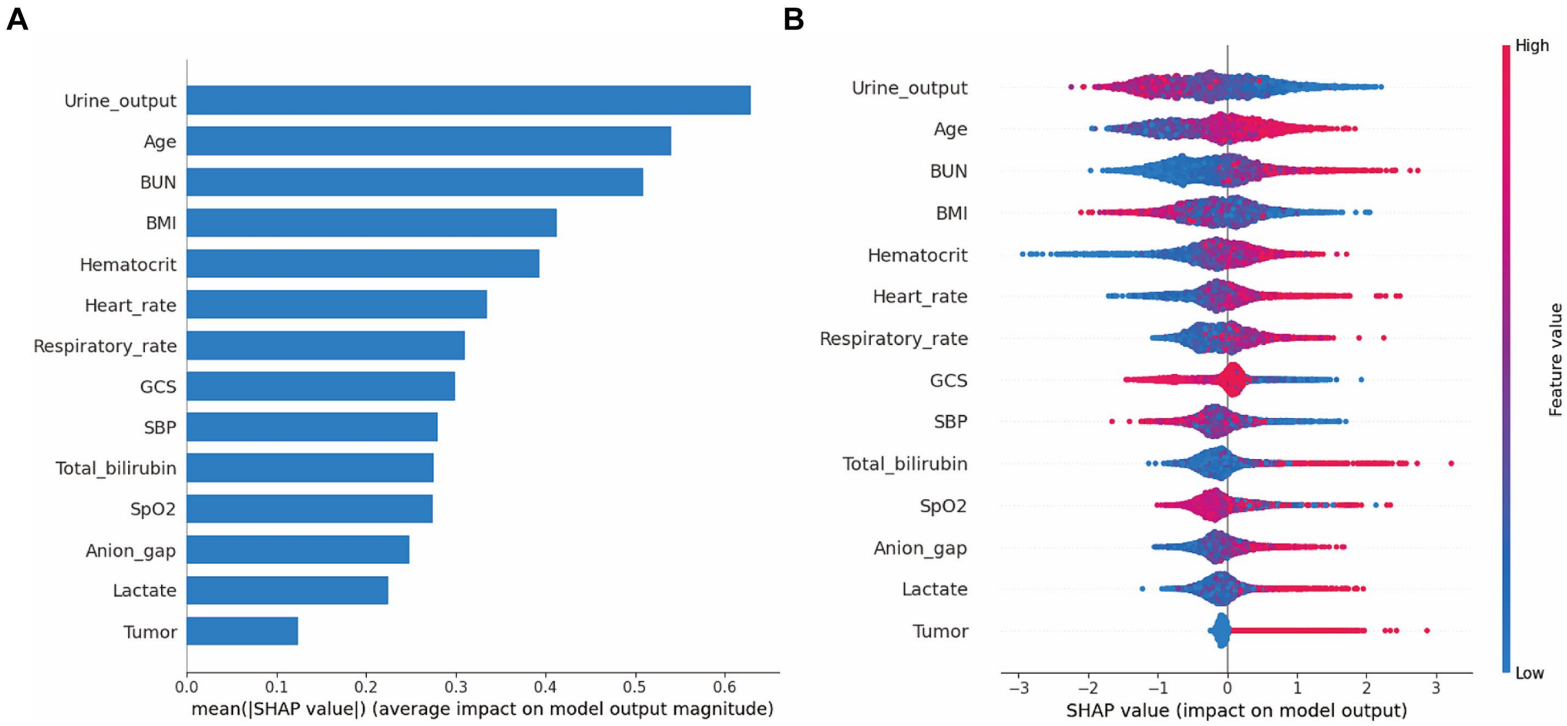
SHAP summary plot for the XGBoost model. The model’s interpretation. **(A)** This plot depicts the feature importance ranking according to the mean (|SHA*p* value|); **(B)** This plot depicts the feature importance based on Shapley values. Herein, red indicates higher feature value, blue indicates lower feature value. Abbreviations: SHAP, Shapley additive explanation; XGBoost, extreme gradient boost; BMI, body mass index; BUN, blood urea nitrogen; GCS, Glasgow Coma Scale; SBP, systolic blood pressure.

**Figure 4:**
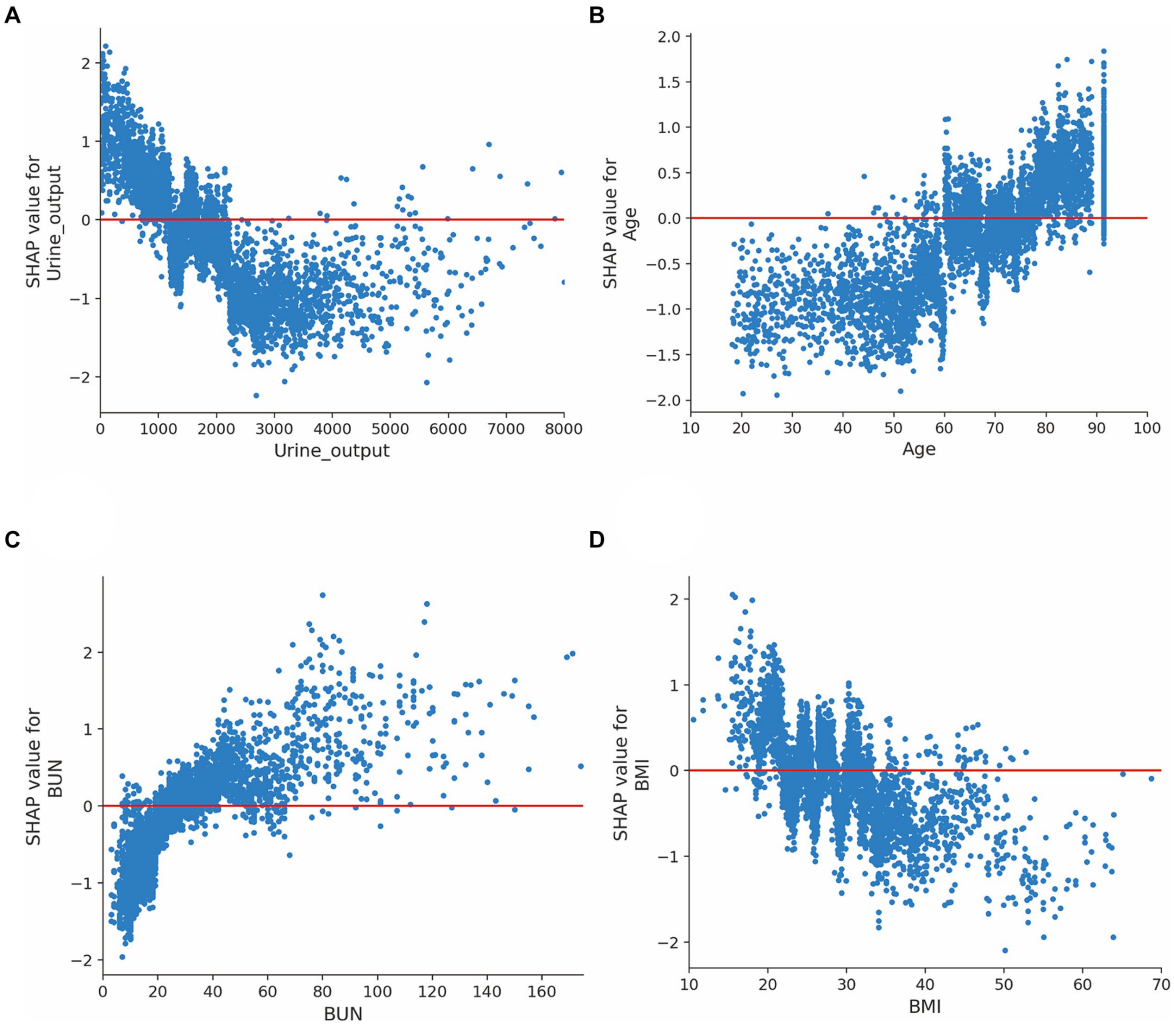
SHAP dependence plot for the XGBoost model. **(A)** Urine output on day 1; **(B)** Age; **(C)** Blood urea nitrogen; **(D)** Body mass index. The y-axis values indicated the SHAP values of features, and the values of features for the x-axis were in the SHAP dependence plot. SHAP values for specific features exceeding zero represent an increased risk of mortality. Abbreviations: SHAP, Shapley additive explanation; XGBoost, extreme gradient boost; BUN, blood urea nitrogen; BMI, body mass index.

Moreover, we evaluated the factors that contributed to the prediction in the XGBoost model. The SHAP summary analysis showed that urine output on day 1, age, BUN and BMI were the top four contributors in the XGBoost model (Figures 3A,B). To better understand the relationship between the four variables and outcome, we displayed the four SHAP dependence plots (Figures 4A–D). These results demonstrated that elevated urine output and BMI were associated with decreased mortality. Reversely, higher levels of BUN and older were associated with increased mortality. Additionally, the SHAP interaction value for XGBoost model is presented in Supplementary Figure S9.

### Model interpretability at the individual level

**Figure 5:**
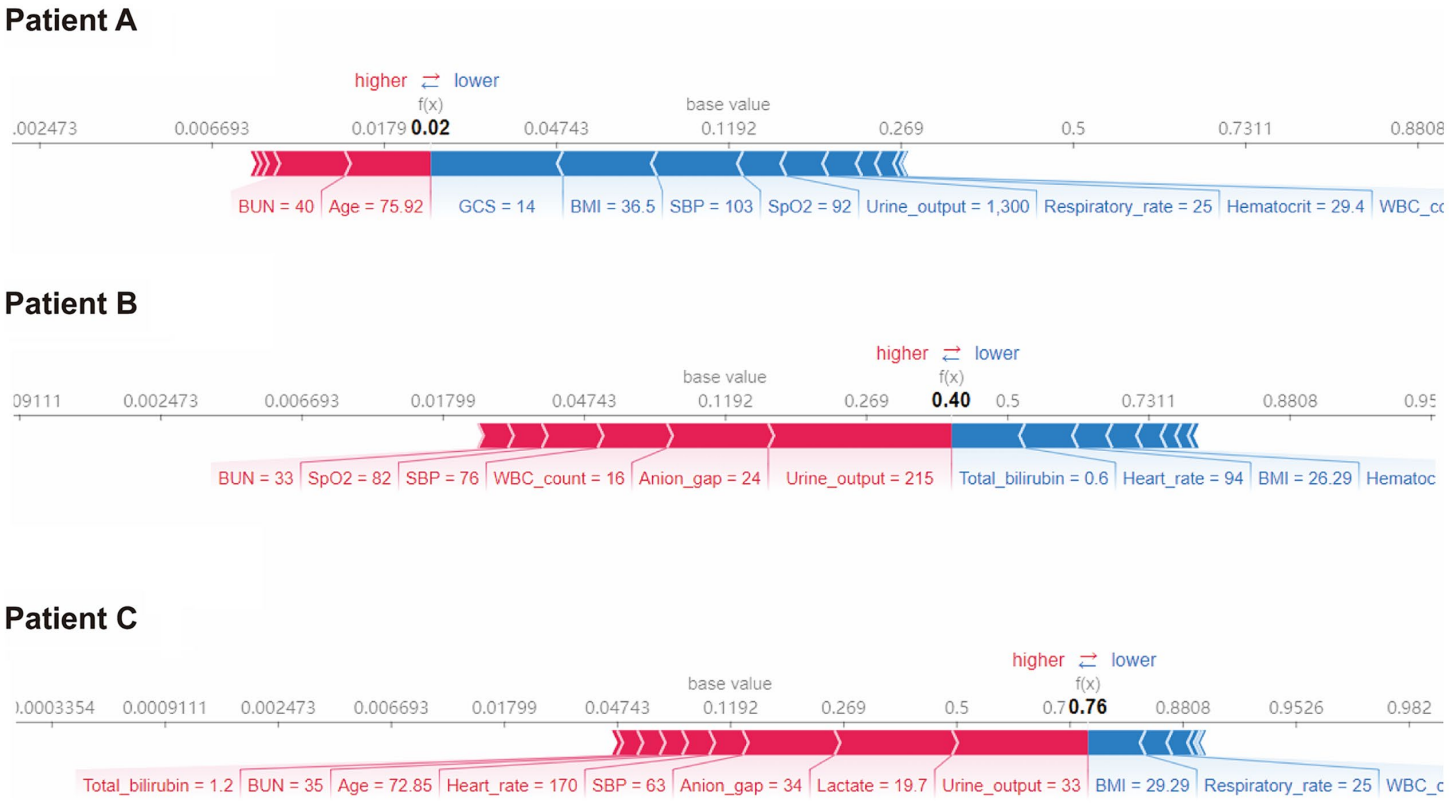
SHAP force plot for the XGBoost model. Characteristic SHAP value influence diagram of three samples. Abbreviations: SHAP, Shapley additive explanation; XGBoost, extreme gradient boost; BMI, body mass index; BUN, blood urea nitrogen; GCS, Glasgow Coma Scale; SBP, systolic blood pressure; WBC, white blood cell.

Furthermore, we conducted SHAP force analysis to illustrate the overall impact of key features on the XGBoost model in three representative individuals (Figure 5). For example, in case-A, the probability of death predicted by the XGBoost model was 2% due to a variety of favorable conditions, consisting of a high GCS value of 14, high BMI value of 36.5 kg/m^2^, high SBP value of 103 mmHg, although a slightly older age (75.9 years) and high BUN value of 40 mg/dL. The true outcome for case-A was survive. In case-B, the probability of death prediction was 40% due to a mixture of conditions, including a low urine output on day 1 (215 mL), high anion gap value of 24 and high WBC count value of 16 × 10^3^/uL, despite a low bilirubin of 0.6 mg/dL. The true outcome for case-B was death. In case-C, the probability of death prediction was relatively high (76%) due to a variety of unfavorable conditions, including a low urine output on day 1 (33 mL), high lactate value of 19.7 mmol/L, despite a high BMI value of 29.29 kg/m^2^. The true outcome for case-C was death.

## Discussion

In this study, we successfully applied ML approaches to develop and validate the prognostic prediction models in patients with sepsis. By comparison, the XGBoost model outperformed RF, LR, SVM and other traditional clinical scoring systems. Moreover, we utilized a latest model interpretation strategy called SHAP to explain how individual risk factors influence mortality in the ML model. These may help to provide clinical decision-making.

Sepsis is a common syndrome in critically ill patients. Early and accurate prediction of prognosis is essential to the clinical decision-making process in patients with sepsis. Notably, several recently randomized clinical trials have demonstrated that use of ML-based disease prediction systems were associated with improved outcomes (Shimabukuro et al., 2017; Escobar et al., 2020). Thus, it is necessary to develop a prognostic prediction model in patients with sepsis.

ML represents the most cost-effective method for model construction, and consequently many computational tools for disease detection and outcome prediction have been recently developed. A recent study conducted by William et al. demonstrated the potential ability of ML models in predicting mortality among sepsis, they found that ML outperformed commonly used clinical risk scores, such as abbMEDS, mREMS and SOFA (van Doorn et al., 2021). Additionally, Taylor and colleagues conducted a retrospective, modeling study and found that ML algorithm was superior to traditional analytic models (MEDS and REMS score) for predicting in-hospital mortality in patients with sepsis (Taylor et al., 2016). Similarly, another modeling study including 923,759 patients also demonstrated the value of ML models in predicting sepsis mortality (Park et al., 2022). Our study supported evidence from the earlier clinical observations that XGBoost model had a superior performance to predict 28-day mortality among patients with sepsis, compared with clinical scoring systems, such as LODS and SOFA score.

The choice between filter and wrapper methods depends on various factors, such as the dataset size, dimensionality, computational resources available, and the specific goals of the analysis (McKearnan et al., 2023). Filter methods like LASSO can be computationally efficient and provide a good balance between feature selection and model building (Yamada et al., 2014). Wrapper methods, while potentially more accurate, might be more computationally expensive and prone to overfitting, especially with high-dimensional datasets. Therefore, we used LASSO to select feature.

To date, the ML techniques still face black box challenges, which making it questionable to implement them in clinical practice (Azodi et al., 2020). Thus, it is essential to ensure transparency for ML and clarify how the ML model works. However, up to now, few studies have used explainable artificial intelligence (XAI) method for model interpretation (The Lancet Respiratory, 2018). In this study, we implemented SHAP method to mitigate the black-box effect of the XGBoost model. The benefits of using SHAP analysis were mainly standing in two aspects. First, it provided an understanding of the impact of input features in the XGBoost model. Second, it provided a mechanism to build trust in the user community of the model by surfacing the features that impacted a particular prediction of the XGBoost model.

In this study, we found that urine output on day 1, age, BUN and BMI were the top four factors that affected the XGBoost model. These risk factors were also reported in previous studies. For example, urine output, a marker for the development of acute kidney injury, is commonly measured in the ICU. A huge study of 161,940 patients identified that lower urine output was independently associated with increased mortality (Heffernan et al., 2022). In line with previous reports, we found that urine output ranked first in the XGBoost model, and we demonstrated that lower urine output was associated with increased mortality among septic patients. BUN is another serum biomarker to evaluate the renal function. Recently, this indicator has been employed in the context of septic patient evaluation because it has been shown to be a key risk factor associated with poor prognosis among patients with sepsis (Li et al., 2021; Han et al., 2022). Consistently, the SHAP analysis in our study also demonstrated that elevated blood urea nitrogen was related to poor outcome in septic patients. Additionally, evidences from previous studies shown that older age were associated with 90-day mortality after adjustment. In our study, we also found that age is another important indicator for mortality in patients with sepsis (Xie et al., 2020; Hu et al., 2022a). Notably, several studies have examined the effects of BMI on mortality with conflicting conclusions. For instance, some studies have observed the lower mortality in the obese (Pepper et al., 2016; Gribsholt et al., 2021), but some researchers believe that elevated BMI was associated with poor outcomes in sepsis (Butler-Laporte et al., 2020; Lin et al., 2020). Recently, the obesity paradox has been found in septic sepsis and several possible reasons that may explain this phenomenon: (1) Excess body fat prevent muscle loss in sepsis; (2) The immunomodulatory role of adiponectin in sepsis (Yeo et al., 2023). Our SHAP analysis also presented that higher BMI was associated with decreased mortality among patients with sepsis.

Compared with previous similar studies, this study has the following differences (Hou et al., 2020; Kong et al., 2020; Hu et al., 2022b; Li et al., 2023). We demonstrated the ability of ML models to predict 28-day mortality in patients with sepsis, highlighting their potential as valuable tools in healthcare. The results indicate the superiority of the proposed approach over previous techniques in sepsis mortality prediction. By leveraging advanced ML algorithms, our models achieved higher accuracy and predictive power, outperforming traditional methods. Additionally, the utilization of the SHAP method improved the transparency of the ML models, providing interpretable insights into the factors influencing the predictions. This enhanced interpretability contributes to clinical decision-making, enabling healthcare professionals to understand the underlying mechanisms and make informed treatment choices for sepsis patients. These findings have significant implications, especially in cases where previous techniques failed to deliver satisfactory results. The proposed approach offers a novel solution that overcomes the limitations of conventional methods, providing more accurate and reliable predictions for sepsis mortality. By highlighting the advantages of our method and referencing situations where other techniques fell short, our study underscores the potential impact of this approach in improving patient outcomes and guiding clinical interventions.

However, this study also has several limitations. First, the MIMIC-III is a single-center database that only included patients in ICU at the Beth Israel Deaconess Medical Center, it remains unknown if the XGBoost model developed from the database applies to outside patients with sepsis. Second, we only extracted the commonly used variables in this database, further exploration was not performed, which may lead to the abandonment of some key variables. Third, the MIMIC-III database consisting of critically ill participants between 2001 and 2012, and there have been many changes in the management of sepsis occurred in the interim. We have attempted to reduce this effect by applying sepsis 3.0 definition. Fourth, the LASSO method used in this study will select one factor randomly when there are two or more highly collinear variables, which therefore reflect the omission of important predictors. Fifth, we only included data in the first 24 h after ICU admission, and ignored the natural progression of sepsis. Sixth, this is a retrospective study, which is only conducted to identify potentially associations and hypothesize about whether identifying septic patients will actually have any benefit. Future studies can focus on validating the developed machine learning models using independent datasets from different healthcare settings or geographical regions. This would help assess the generalizability and robustness of the models across diverse patient populations and healthcare systems.

## Conclusion

Our study successfully demonstrated the effectiveness of ML models in predicting 28-day mortality in patients with sepsis. The results showcase the potential of ML as a valuable tool in healthcare, offering accurate predictions for clinical outcomes. Furthermore, the application of the SHAP method enhanced the transparency of the ML model, providing interpretable insights into the factors influencing the predictions. This increased transparency has significant implications for clinical decision-making, enabling healthcare professionals to better understand and trust the model’s output. Overall, the combination of ML models and the SHAP method holds promise for improving patient care and outcomes in the context of sepsis management.

## Data availability statement

The original contributions presented in the study are included in the article/Supplementary material, further inquiries can be directed to the corresponding author.

## Ethics statement

Ethical approval was not required for the study involving human data in accordance with the local legislation and institutional requirements. Written informed consent to participate in this study was not required from the participants in accordance with the national legislation and the institutional requirements.

## Author contributions

BH: Conceptualization, Data curation, Formal analysis, Investigation, Methodology, Software, Validation, Visualization, Writing – original draft, Writing – review & editing. ZQ: Conceptualization, Formal analysis, Methodology, Project administration, Resources, Supervision, Validation, Visualization, Writing – original draft, Writing – review & editing.
