# Supplementary material for: Development and validation of an interpretable machine learning for mortality prediction in patients with sepsis
Source: Front Artif Intell. 2024 Jul 8;7:1348907. doi: 10.3389/frai.2024.1348907 (PMC11262051; doi:10.3389/frai.2024.1348907)
Supplement: Supplementary file 2 [file Data_Sheet_1.docx]

**Development and validation of an interpretable machine learning for mortality prediction in patients with sepsis**

**Supplementary Note**

We calculated the maximum value of the Logistic Organ Dysfunction Score (LODS) and Sequential Organ Failure Assessment (SOFA) on the first day of each septic patients' stay [1,2].

Reference:

[1] Le Gall JR, Klar J, Lemeshow S, et al. The Logistic Organ Dysfunction system. A new way to assess organ dysfunction in the intensive care unit. ICU Scoring Group. JAMA. 1996;276(10):802-810.

[2] Vincent JL, Moreno R, Takala J, et al. The SOFA (Sepsis-related Organ Failure Assessment) score to describe organ dysfunction/failure. On behalf of the Working Group on Sepsis-Related Problems of the European Society of Intensive Care Medicine. Intensive Care Med. 1996;22(7):707-710.

**The components of SOFA score.**

| **Variable** | **Index** | **0** | **1** | **2** | **3** | **4** |
| --- | --- | --- | --- | --- | --- | --- |
| **SOFA_res** | PaO_2_/FiO_2_ (mmHg) | ≥400 | <400 | <300 | <200 | <100 |
| **SOFA_coag** | Platelets, x 10^3^/uL | ≥150 | <150 | <100 | <50 | <20 |
| **SOFA_liver** | Bilirubin, mg/dL | <1.2 | 1.2-1.9 | 2.0-5.9 | 6.0-11.9 | >12.0 |
| **SOFA_ner** | Glasgow coma score | 15 | 13-14 | 10-12 | 6-9 | <6 |
| **SOFA_renal** | Creatinine, mg/dL | <1.2 | 1.2-1.9 | 2.0-3.4 | 3.5-4.9 | ≥5.0 |
|  | Urine Output, ml/d | ≥500 |  |  | <500 | <200 |
| **SOFA_vas** | MAP, mmHg | ≥70 | <70 |  |  |  |
|  | Dopamine, ug/kg/min |  |  | ≤5 | >5 | >15 |
|  | Dobutamine |  |  | Any |  |  |
|  | Epinephrine, ug/kg/min |  |  |  | ≤0.1 | >0.1 |
|  | Norepinephrine, ug/kg/min |  |  |  | ≤0.1 | >0.1 |

**The components of LODS score.**

| **Variable** | **Index** | **5** | **3** | **1** | **0** | **1** | **3** | **5** |
| --- | --- | --- | --- | --- | --- | --- | --- | --- |
| **LODS_ner** | Glasgow coma score | 3-5 | 6-8 | 9-13 | 14-15 |  |  |  |
| **LODS_cardio** | Heart rate, beats/min | <30 |  |  | 30-139 | ≥140 |  |  |
|  | Systolic blood pressure, mmHg | <40 | 40-69 | 70-89 | 90-239 | 240-269 | ≥270 |  |
| **LODS_renal** | Serum urea, g/L |  |  |  | <0.36 | 0.36-0.59 | 0.60-1.19 | ≥1.20 |
|  | Serum urea nitrogen, mg/dL |  |  |  | <17 | 17-28 | 28-56 | ≥56 |
|  | Creatinine, mg/dL |  |  |  | <1.20 | 1.20-1.59 | ≥1.60 |  |
|  | Urine output, L/d | <0.5 | 0.5-0.74 |  | 0.75-9.99 |  |  |  |
| **LODS_lung** | PaO_2_/FiO_2_, mmHg |  | <150 | ≥150 |  |  |  |  |
| **LODS_hema** | White blood cell count, x 10^9^/L |  | <1.0 | 1.0-2.4 | 2.5-49.9 | ≥ 50.0 |  |  |
|  | Platelets, x 10^9^/L |  |  | <50 | ≥50 |  |  |  |
| **LODS_hepa** | Bilirubin, mg/dL |  |  |  | <2.0 | ≥2.0 |  |  |
|  | Prothrombin time, s |  |  |  | ≤3 | ＞3 |  |  |

**Online Data Supplement**

**• Table S1. All variables extracted from the MIMIC-III database.**

**•** **Table S2. The candidate variables for model development.**

**• Table S3. Baseline characteristics between training set and testing set.**

**• Table S4. The key hyperparameters for each machine learning model.**

**• Figure S1. The timeline of the sepsis definition.**

**•** **Figure S2. The screening flowchart.**

**• Figure S3. The distribution plot of mortality days.**

**• Figure S4. The fraction of missing values for all extracted features.**

**• Figure S5. The LASSO regression for feature selection.**

**• Figure S6. Correlation matrix of the candidate features.**

**• Figure S7. ROC curves in testing set.**

**• Figure S8. The calibration curve for the XGBoost model.**

**• Figure S9. SHAP interaction value for XGBoost model.**

**Table S1. All variables extracted from the MIMIC-III database.**

| Age | **Demographic characteristics (3)** |
| --- | --- |
| Gender |  |
| Body mass index |  |
| Hypertension | **Comorbidities (7)** |
| Diabetes |  |
| Congestive heart failure |  |
| Renal failure |  |
| Liver disease |  |
| Tumor |  |
| Rheumatoid arthritis |  |
| Heart rate | **Vital signs (7)** |
| Respiratory rate |  |
| Systolic blood pressure |  |
| Diastolic blood pressure |  |
| Mean artery pressure |  |
| Body temperature |  |
| SpO_2_ |  |
| Glucose | **Laboratory results (22)** |
| Lactate |  |
| White blood cell count |  |
| Platelet |  |
| Hematocrit |  |
| Hemoglobin |  |
| Aspartate aminotransferase |  |
| Alanine aminotransferase |  |
| Creatine kinase MB |  |
| Lactate dehydrogenase |  |
| Total bilirubin |  |
| Albumin |  |
| Prothrombin time |  |
| Partial thromboplastin time |  |
| International normalized ratio |  |
| Creatinine |  |
| Blood urea nitrogen |  |
| Sodium |  |
| Chloride |  |
| Potassium |  |
| Bicarbonate |  |
| Anion gap |  |
| Urine output | **Urine output on day 1 (1)** |
| Glasgow Coma Scale | **Severity of illness score (1)** |

**Table S2. The candidate variables for model development.**

| Age | **Demographic characteristics (2)** |
| --- | --- |
| Body mass index |  |
| Tumor | **Comorbidity (1)** |
| Heart rate | **Vital signs (4)** |
| Respiratory rate |  |
| Systolic blood pressure |  |
| SpO_2_ |  |
| Lactate | **Laboratory results (5)** |
| Hematocrit |  |
| Total bilirubin |  |
| Blood urea nitrogen |  |
| Anion gap |  |
| Urine output | **Urine output on day 1 (1)** |
| Glasgow Coma Scale | **Severity of illness score (1)** |

**Table S3. Baseline characteristics between training set and testing set.**

| **Characteristics** | **Total**  **(*N* = 5834)** | **Training set**  **(*N* = 4375)** | **Testing set**  **(*N* = 1459)** | ***P* value** |
| --- | --- | --- | --- | --- |
| **Demographics** |  |  |  |  |
| Age, year | 66(54-78) | 67(54-78) | 66(53-77) | 0.274 |
| Gender |  |  |  | 0.113 |
| Male, n (%) | 3492(59.9) | 2593(59.3) | 899(61.6) |  |
| Female, n (%) | 2342(40.1) | 1782(40.7) | 560(38.4) |  |
| Body mass index, kg/m^2^ | 27.6(24.2-31.9) | 27.6(24.2-31.9) | 27.7(24.2-31.9) | 0.915 |
| **Comorbidities** |  |  |  |  |
| Diabetes, n (%) | 1557(26.7) | 1175(26.9) | 382(26.2) | 0.614 |
| Congestive heart failure, n (%) | 878(15.0) | 674(15.4) | 204(14.0) | 0.188 |
| Renal failure, n (%) | 802(13.7) | 620(14.2) | 182(12.5) | 0.103 |
| Hypertension, n (%) | 697(11.9) | 541(12.4) | 156(10.7) | 0.088 |
| Liver disease, n (%) | 377(6.5) | 279(6.4) | 98(6.7) | 0.648 |
| Tumor, n (%) | 369(6.3) | 280(6.4) | 89(6.1) | 0.684 |
| Rheumatoid arthritis, n (%) | 153(2.6) | 128(2.9) | 25(1.7) | 0.012 |
| **Vital signs on day 1** |  |  |  |  |
| Heart rate, bpm | 104(91-118) | 104(91-118) | 104(91-118) | 0.829 |
| Systolic blood pressure, mmHg | 89(79-99) | 89(79-98) | 89(80-99) | 0.720 |
| Diastolic blood pressure, mmHg | 43(37-50) | 43(37-50) | 43(37-50) | 0.274 |
| Mean arterial pressure, mmHg | 57(51-64) | 58(51-64) | 57(51-64) | 0.857 |
| Respiratory rate | 27(23-31) | 27(23-31) | 26(23-31) | 0.277 |
| Body temperature, ℃ | 37.6(37-38.2) | 37.6(37.1-38.2) | 37.6(37.1-38.1) | 0.945 |
| SpO_2_, % | 93(90-95) | 93(90-95) | 93(91-95) | 0.848 |
| **Laboratory findings on day 1** |  |  |  |  |
| Blood glucose, mg/dL | 170(139-212) | 170(139-213) | 169(139-206) | 0.467 |
| Lactate, mmol/L | 2.3(1.5-3.4) | 2.3(1.5-3.4) | 2.3(1.6-3.4) | 0.212 |
| White blood cell count, ×10^3^/uL | 13.6(10.1-18.2) | 13.6(10.1-18.3) | 13.3(10.1-17.8) | 0.306 |
| Platelets, ×10^3^/uL | 170(119-232) | 169(119-231) | 171(119-233) | 0.576 |
| Hematocrit, % | 29(25-33) | 29(25-33) | 29(25-33) | 0.546 |
| Hemoglobin, g/dL | 9.8(8.4-11.2) | 9.8(8.4-11.2) | 9.8(8.5-11.3) | 0.450 |
| Total bilirubin, mg/dL | 0.7(0.4-1.4) | 0.7(0.4-1.4) | 0.7(0.4-1.4) | 0.715 |
| Prothrombin time, s | 14.7(13.4-16.8) | 14.8(13.5-16.9) | 14.7(13.4-16.7) | 0.130 |
| Partial thromboplastin time, s | 33(28-45) | 33(28-45) | 33(27-45) | 0.169 |
| International normalized ratio | 1.3(1.2-1.6) | 1.3(1.2-1.6) | 1.3(1.2-1.6) | 0.160 |
| Creatinine, mg/dL | 1.1(0.8-1.6) | 1.1(0.8-1.6) | 1.1(0.8-1.6) | 0.994 |
| Blood urea nitrogen, mg/dL | 21(15-35) | 21(15-35) | 21(15-35) | 0.852 |
| Sodium, mmol/L | 141(138-143) | 141(138-143) | 141(138-143) | 0.506 |
| Chloride, mmol/L | 109(105-112) | 109(105-112) | 109(105-112) | 0.737 |
| Potassium, mmol/L | 4.6(4.2-5.3) | 4.6(4.1-5.2) | 4.7(4.2-5.3) | 0.083 |
| Bicarbonate, mmol/L | 25(22-27) | 25(22-27) | 25(22-27) | 0.760 |
| Anion gap | 15(12-18) | 15(12-18) | 15(12-17) | 0.638 |
| **Urine output on day 1, mL** | 1724(1088-2540) | 1725(1095-2550) | 1715(1065-2525) | 0.505 |
| **Severity of illness scores** |  |  |  |  |
| GCS | 15(14-15) | 15(14-15) | 15(14-15) | 0.733 |
| SOFA | 5(3-7) | 5(3-7) | 5(3-7) | 0.997 |
| LODS | 4(3-7) | 4(3-7) | 4(3-7) | 0.671 |

Data were reported as no. (%) or median (IQR).

Abbreviations: GCS, Glasgow Coma Scale; SOFA, Sequential Organ Failure Assessment; LODS, Logistic Organ Dysfunction System.

**Table S4. The key hyperparameters for each machine learning model.**

| Models | Hyperparameters |
| --- | --- |
| XGBoost | Estimators=100  Gamma=0.0  Max depth=20  Min child weight=0.1  Learning rate=0.1  Subsample=0.5 |
| Random forest | Estimators=80  Max depth=5 |
| Support vector machine | Kernel=rbf  C=1.0  Gamma=0.04 |
| Logistic regression | C=0.001  Penalty=l2 |

**Figure S1. The timeline of the sepsis definition.**


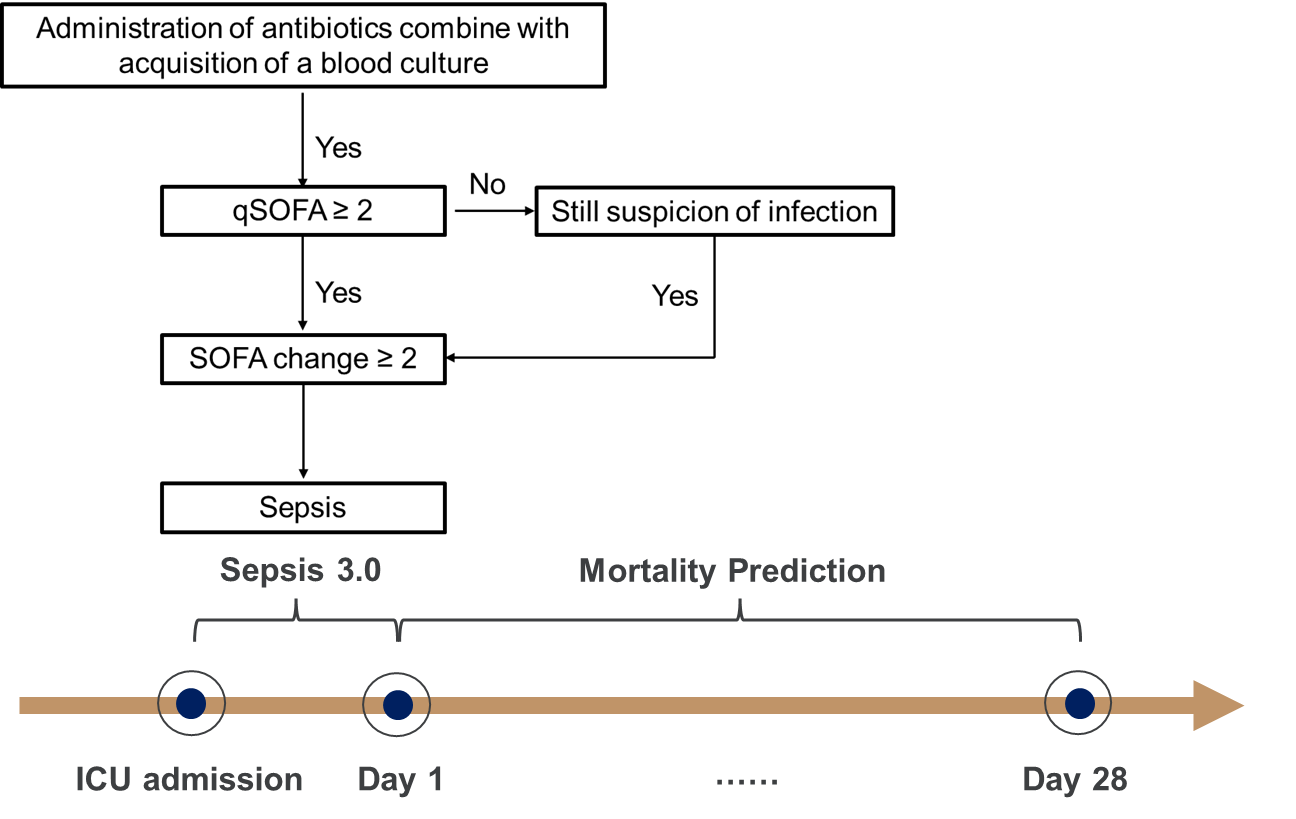


|  | **With unknown baseline organ dysfunction** | **With baseline organ dysfunction** |
| --- | --- | --- |
| **SOFA_baseline** | 0 | SOFA_min |
| **SOFA_max** | SOFA_max | SOFA_max |
| **SOFA_change** | SOFA_max | SOFA_max - SOFA_min |

SOFA_max: the maximum SOFA value on the first day of ICU admission

SOFA_min: the minimum SOFA value on the first day of ICU admission

Note: Our calculations focused solely on the baseline organ dysfunction in SOFA_min, rather than considering complications.

Abbreviations: SOFA, Sequential Organ Failure Assessment; ICU, intensive care unit.

**Figure S2. The screening flowchart.**


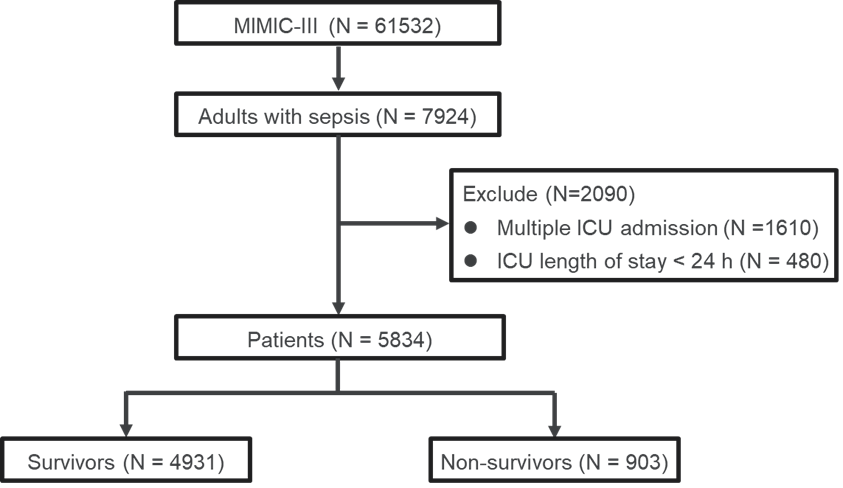


Abbreviations: MIMIC-III, the Medical Information Mart for Intensive Care III; ICU, intensive care unit.

**Figure S3. The distribution plot of mortality days.**


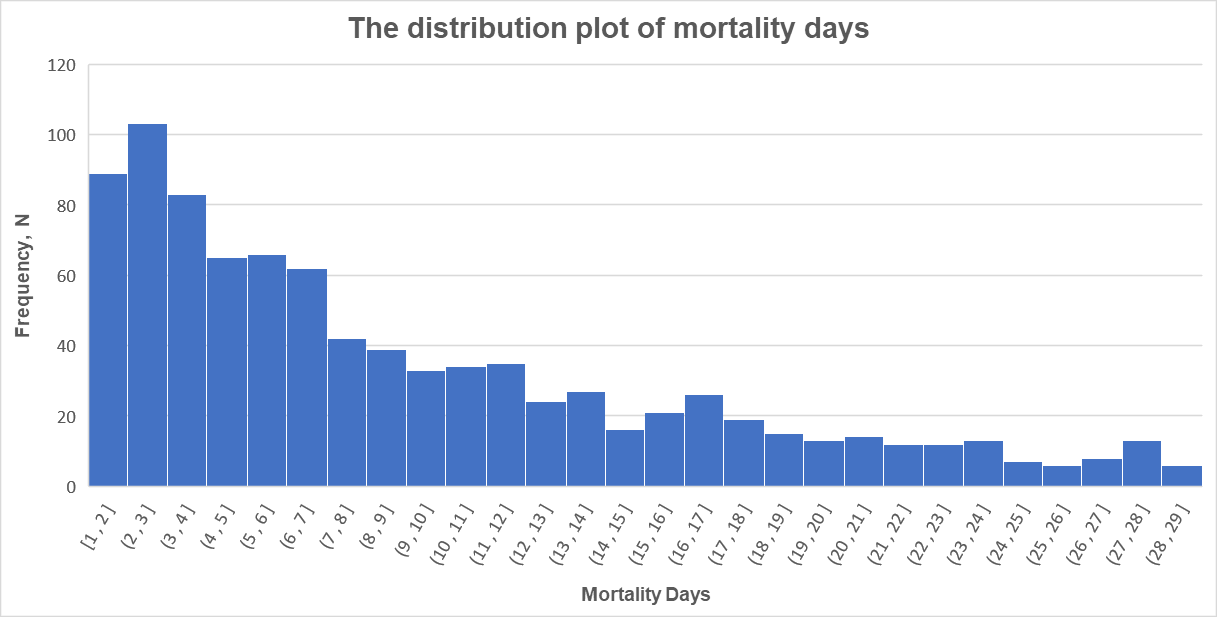


**Figure S4.** **The fraction of missing values for all extracted features.**

**
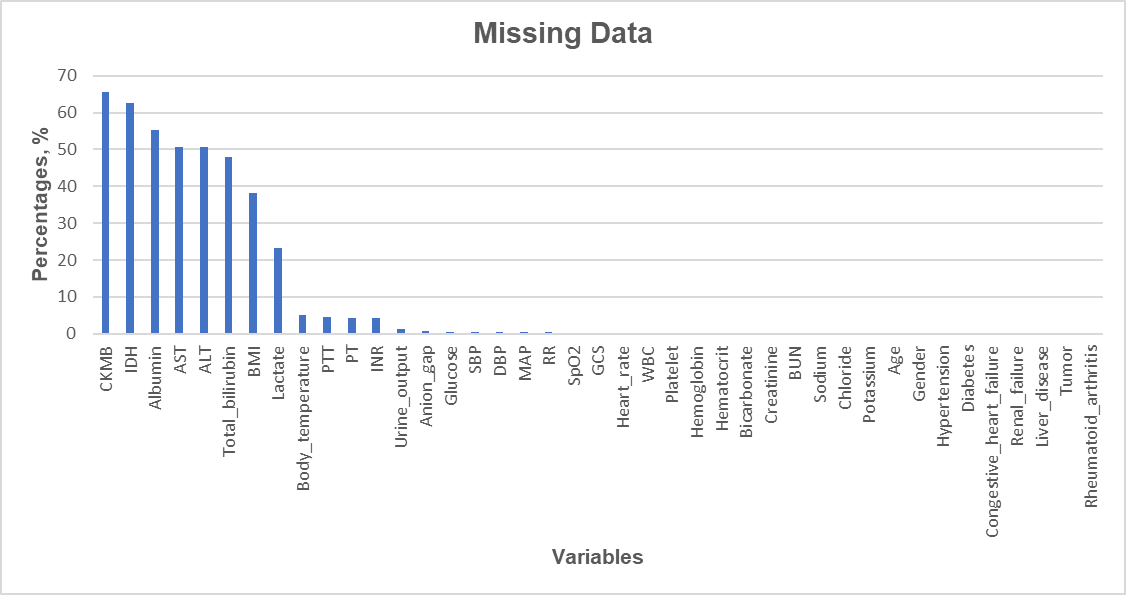
**

Variables with missingness > 50% were removed.

**Figure S5. The LASSO regression for feature selection.**

**
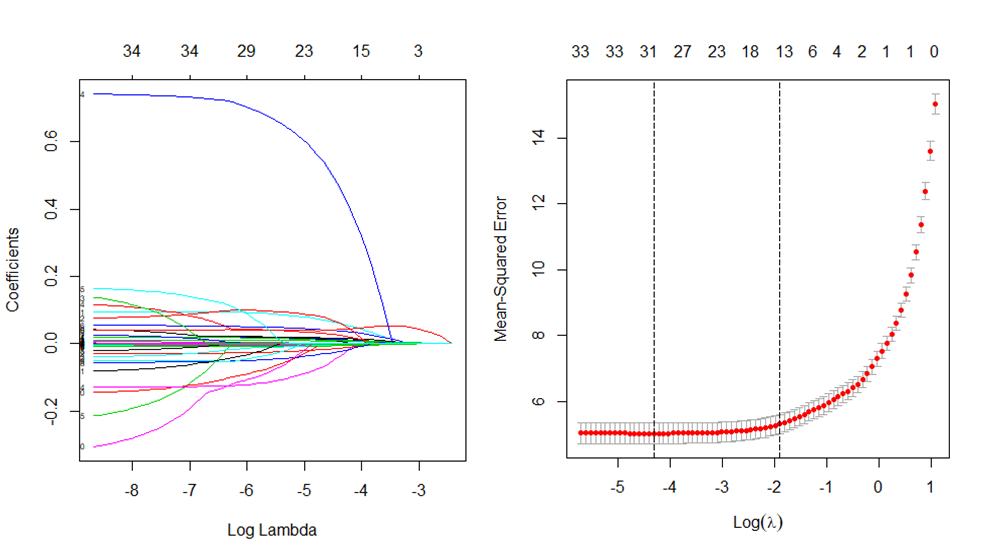
A B**

1. **The optimal parameter (lambda) selection in the LASSO model involved using 5-fold cross-validation with the minimum criteria.**

We plotted the curve of the partial likelihood deviance (binomial deviance) against log (lambda). Dotted vertical lines were drawn at the optimal values determined by employing the minimum criteria and the 1 SE of the minimum criteria.

1. **The LASSO coefficient profiles of the full variables.**

We generated a coefficient profile plot along the log (lambda) sequence. 14 variables with nonzero coefficients were selected based on the optimal lambda.

Abbreviations: LASSO, least absolute shrinkage and selection operator.

**Figure S6. Correlation matrix of the candidate features.**


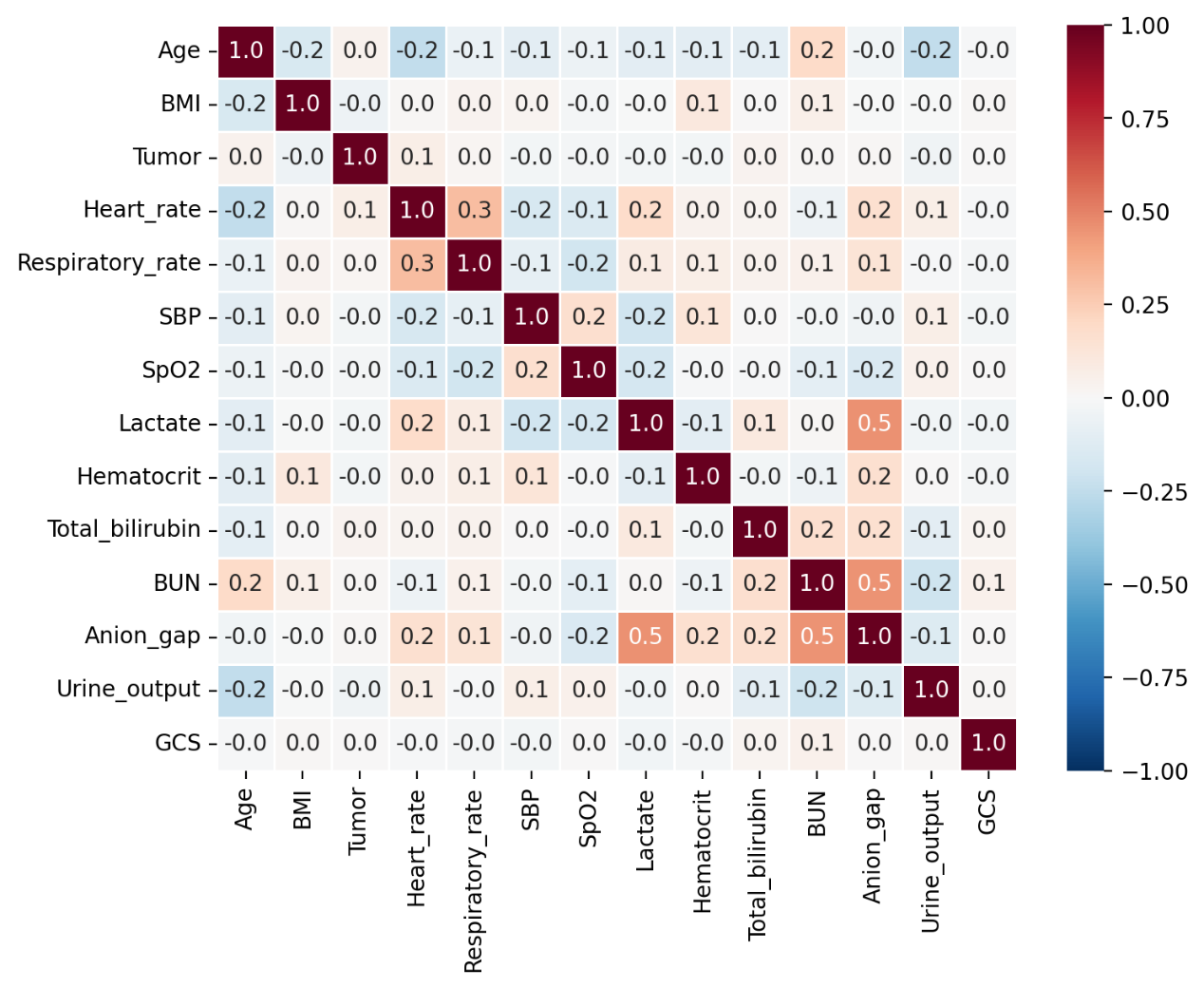


Coefficients are derived using the Spearman’s rank correlation coefficient.

Abbreviations: BMI, body mass index; SBP, systolic blood pressure; BUN, blood urea nitrogen; GCS, Glasgow Coma Scale.

**Figure S7. ROC curves in testing set.**


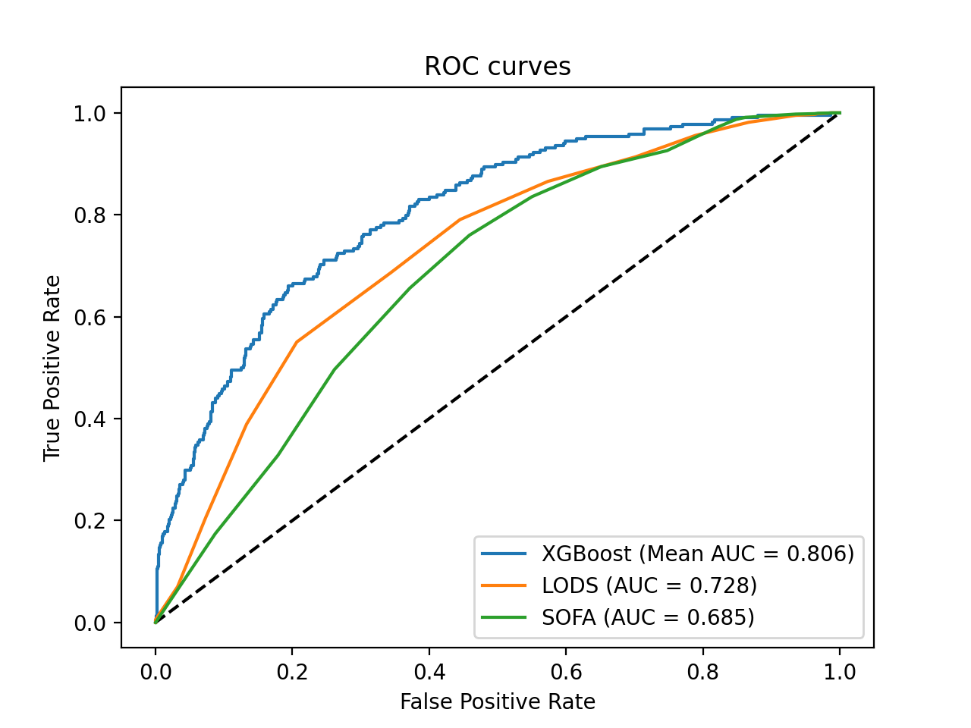


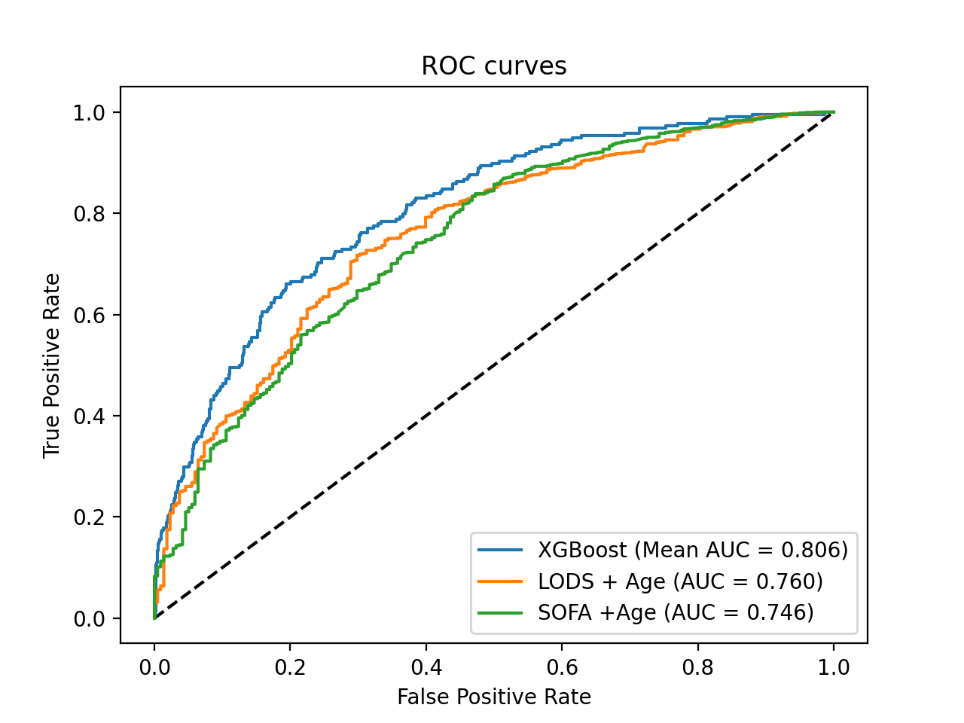


Considering the lack of age in SOFA and LODS components, we compared the AUC performance between XGBoost model and SOFA+age and LODS+age in predicting 28-day mortality in testing set.

Abbreviations: ROC, receiver operating characteristic curve; AUC, area under receiver operator characteristic curve; SOFA, Sequential Organ Failure Assessment; LODS, Logistic Organ Dysfunction System.

**Figure S8. The calibration curve for the XGBoost model.**

**
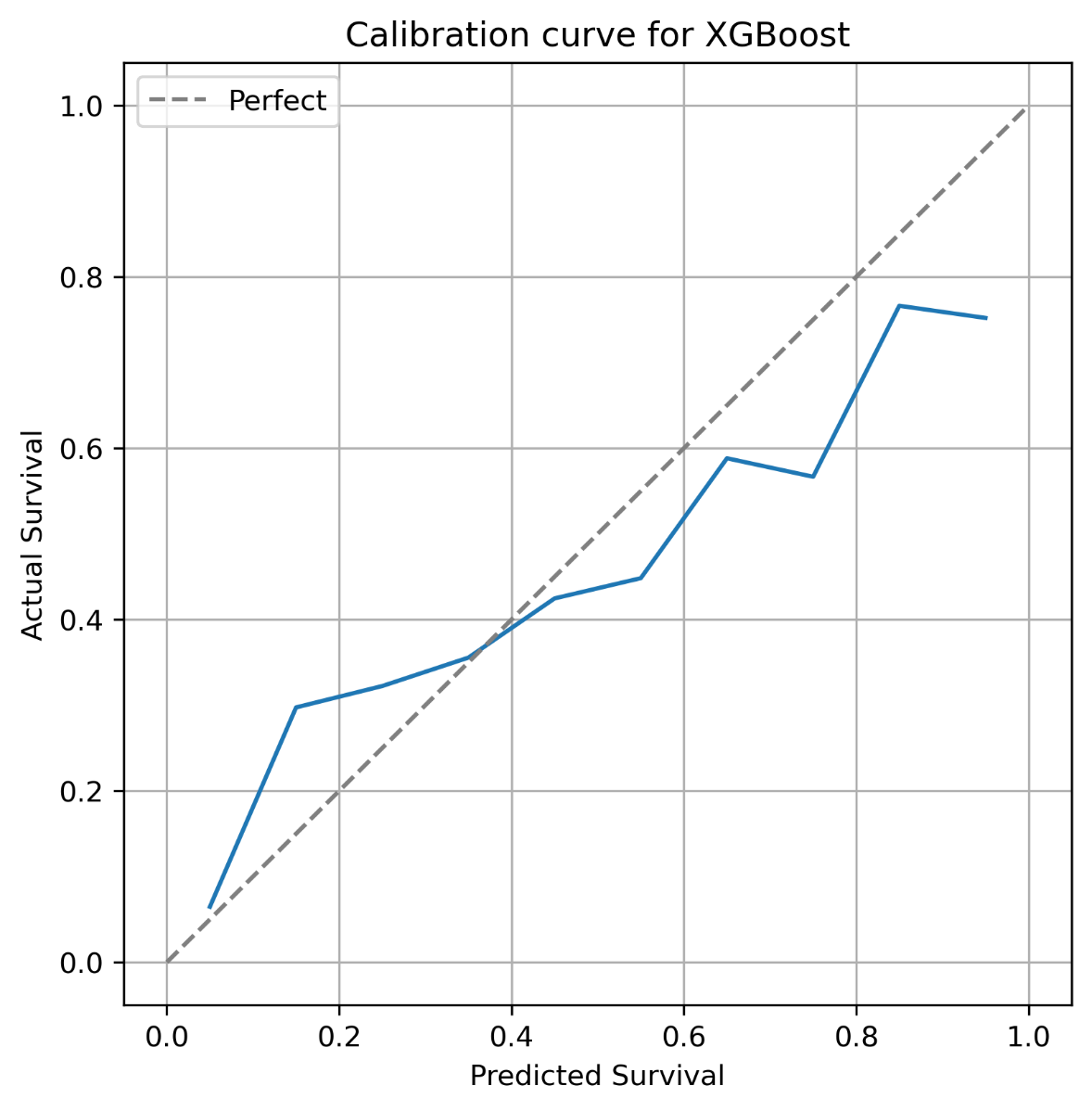
**

Model evaluation: The chi-score value of Hosmer-Lemeshow goodness of fit test is 8.216 (*P* = 0.354 > 0.05), the Brier score is 0.05. The calibration curve shows that the calibration of the model is reasonable.

Abbreviations: XGBoost, extreme gradient boost.

**Figure S9. SHAP interaction value for XGBoost model.**


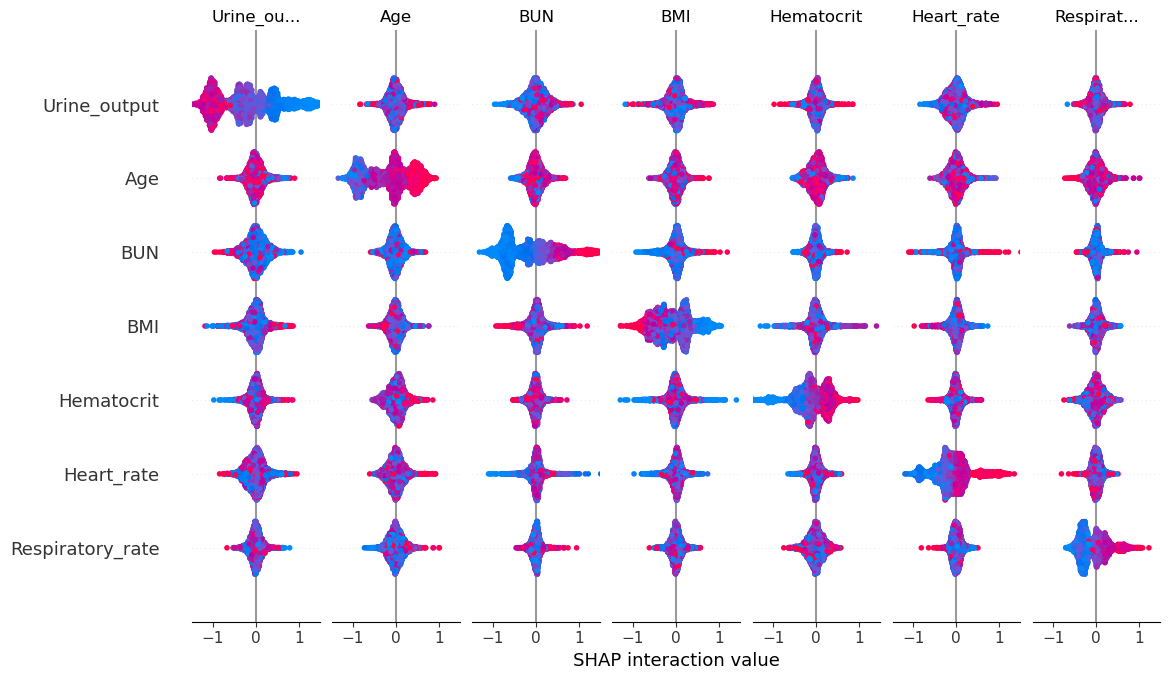


SHAP interaction values are a generalization of SHAP values to higher order interactions. Fast exact computation of pairwise interactions are implemented for tree models with shap.TreeExplainer(model). This returns a matrix for every prediction, where the main effects are on the diagonal and the interaction effects are off-diagonal.

Abbreviations: SHAP, Shapley additive explanation; XGBoost, extreme gradient boost; BMI, body mass index; BUN, blood urea nitrogen; WBC, white blood cell count; GCS, Glasgow Coma Scale.
